# Do Characteristics of Faces That Convey Trustworthiness and Dominance Underlie Perceptions of Criminality?

**DOI:** 10.1371/journal.pone.0037253

**Published:** 2012-06-04

**Authors:** Heather D. Flowe

**Affiliations:** College of Medicine, Biological Sciences and Psychology, University of Leicester, Leicester, United Kingdom; Tel Aviv University, Israel

## Abstract

**Background:**

This study tested whether the 2D face evaluation model proposed by Oosterhof and Todorov can parsimoniously account for why some faces are perceived as more criminal-looking than others. The 2D model proposes that trust and dominance are spontaneously evaluated from features of faces. These evaluations have adaptive significance from an evolutionary standpoint because they indicate whether someone should be approached or avoided.

**Method:**

Participants rated the emotional state, personality traits, and criminal appearance of faces shown in photographs. The photographs were of males and females taken under naturalistic conditions (i.e., police mugshots) and highly controlled conditions. In the controlled photographs, the emotion display of the actor was systematically varied (happy expression, emotionally neutral expression, or angry expression).

**Results:**

Both male and female faces rated high in criminal appearance were perceived as less trustworthy and more dominant in police mugshots as well as in photographs taken under highly controlled conditions. Additionally, emotionally neutral faces were deemed as less trustworthy if they were perceived as angry, and more dominant if they were morphologically mature. Systematically varying emotion displays also affected criminality ratings, with angry faces perceived as the most criminal, followed by neutral faces and then happy faces.

**Conclusion:**

The 2D model parsimoniously accounts for criminality perceptions. This study extends past research by demonstrating that morphological features that signal high dominance and low trustworthiness can also signal high criminality. Spontaneous evaluations regarding criminal propensity may have adaptive value in that they may help us to avoid someone who is physically threatening. On the other hand, such evaluations could inappropriately influence decision making in criminal identification lineups. Hence, additional research is needed to discover whether and how people can avoid making evaluations regarding criminality from a person’s facial appearance.

## Introduction

This study aimed to understand how we make judgments of criminality from facial appearance. People have well defined stereotypes about what criminal perpetrators look like [1], [2], [3], [4]–[5]. People who commit crime are thought to have long or shaggy dark hair, tattoos, beady eyes, pock marks and scars [3]. Faces rated high in cri[minal appearance are also more likely to be remembered [4], (c.f. [6]) and identified from police lineups based on guessing alone [7], a tendency called *criminal face bias*. Additionally, when asked to make a global assessment on a Likert-type scale regarding the extent to which a face appears criminal, reliability across raters is high [4], [7]. However, despite widespread agreement concerning whether a given face looks criminal, the question of how these inferences are made remains unanswered. A parsimonious account of the factors that underlie criminal facial appearance would have enormous theoretical and applied utility. For example, if we identified the factors that make people seem criminal-looking, it may be possible to hold these factors constant across members of a lineup, thereby reducing the impact of criminal face bias on lineup identifications.

According to the *emotion overgeneralization hypothesis*, people infer personality traits from the similarity of a person’s morphological facial features to emotional expressions [8]. Emotionally neutral faces are rated as having an angry appearance when the distance between the eyes and the mouth is relatively short [9]. A relatively short distance between the eyes and the mouth mimics an anger display, wherein the mouth is raised upward toward the eyes. Emotionally neutral faces that appear angry are perceived higher in dominance compared to their counterparts, and emotionally neutral faces that appear happy are perceived as trustworthy [10]. Additionally, morphologically mature faces are viewed as physically stronger, more dominant, and less honest, kind, and warm compared to morphologically baby faces [11], [12], [13], [14]. Personality traits are also inferred from transient emotional displays. Faces momentarily expressing happiness are perceived as having positive social traits whereas faces expressing anger are perceived as being socially dominant [15].

Recently, Oosterhof and Todorov proposed a unifying framework for face evaluation [16]. They accumulated strong evidence that spontaneous trait inferences made on the basis of facial appearance arise from just two fundamental dimensions: valence and dominance. In their 2D model of face evaluation, the valence dimension signals whether a person should be approached or avoided, whereas the dominance dimension signals how capable a person is of inflicting physical harm. Features of faces that suggest valence, such as happiness and anger, are overgeneralised to evaluate whether a person is trustworthy and should be approached or avoided. Features of faces that suggest dominance, such as masculinity and maturity, are overgeneralized to evaluate physical strength. Oosterhof and Todorov proposed that these judgments have adaptive value–they serve as social signals as to whether a person should be approached or avoided and how capable a person is of causing us physical harm. In support of this proposition, they found that assessments of threat derived from facial appearance are negatively associated with perceptions of trustworthiness and positively associated with perceptions of dominance.

The purpose of the present study was to assess whether the 2D face evaluation model can account for perceptions of criminality that arise from facial appearance. The 2D model was deemed ideal for this purpose because of its potential to provide a parsimonious account of why some faces are viewed as more criminal-looking than others. Based on the model, it was hypothesized that faces perceived as criminal-looking would appear threatening, less trustworthy and more dominant. Additionally, perceptions of trustworthiness and dominance were expected to vary in relation to the morphological features of faces. Inferences regarding trustworthiness were expected to vary with the emotion perceived from a face. Angrier faces were predicted to be judged as less trustworthy and more criminal in appearance. Perceptions of dominance were expected to vary in relation to face maturity, with morphologically mature-faced individuals judged as more dominant and more criminal-looking.

**Table 1:** Reliability (Cronbach’s α) for Emotional State, Trait and Criminality Ratings for the Controlled and Naturalistic Photos by Gender.

*Controlled*		
	Male	Female
Angry	0.92 (*n* = 16)	0.89 (*n* = 16)
Dominant	0.88 (*n* = 16)	0.88 (*n* = 16)
Mature	0.94 (*n* = 16)	0.91 (*n* = 16)
Threatening	0.90 (*n* = 16)	0.91 (*n* = 16)
Trustworthy	0.91 (*n* = 16)	0.88 (*n* = 16)
Criminal (neutral pose)	0.91 (*n* = 16)	0.89 (*n* = 16)
Criminal (happy pose)	0.96 (*n* = 16)	0.94 (*n* = 16)
Criminal (angry pose)	0.97 (*n* = 16)	0.96 (*n* = 16)
***Naturalistic***		
	**Male**	**Female**
Angry	0.94 (*n* = 22)	0.93 (*n* = 22)
Dominant	0.96 (*n* = 20)	0.95 (*n* = 20)
Mature	0.95 (*n* = 22)	0.93 (*n* = 22)
Threatening	0.97 (*n* = 20)	0.95 (*n* = 20)
Trustworthy	0.97 (*n* = 20)	0.96 (*n* = 20)
Criminal	0.98 (*n* = 24)	0.96 (*n* = 24)

These predictions were tested using photographs of men and women that were taken in naturalistic and controlled settings. In the naturalistic sample, participants rated the attributes of people portrayed in police mugshots. The naturalistic sample provided the opportunity to test whether the 2D model may be generalised to an applied setting in which person perception processes may have grave consequences. In the controlled sample, people rated the emotion, personality attributes, and criminal appearance of actors’ faces when in an emotionally neutral pose, and these ratings were analysed to further test whether perceptions of criminality are underpinned by assessments of trustworthiness and dominance. Additionally, the criminal appearance of the actors’ faces when in an angry, a happy, or a neutral emotional expression was also measured to assess the effect of angry expression on criminality perceptions.

**Figure 1:**
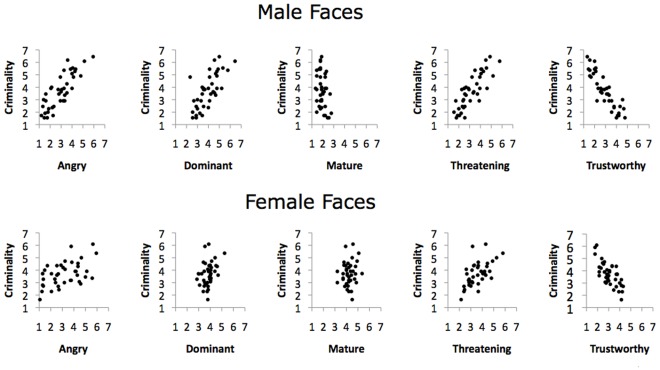
Scatterplots illustrating the bivariate relationship of criminality with the other attributes measured for the naturalistic photos by face gender.

**Table 2:** Pearson’s Bivariate Correlation Coefficients for Male and Female Faces, Naturalistic Photographs.

Male Faces					
	*Criminal*	*Threatening*	*Trustworthy*	*Dominant*	*Angry*
*Threatening*	.83**				
*Trustworthy*	−.89**	−.82**			
*Dominant*	.73**	.75**	−.69**		
*Angry*	.83**	.88**	−.88**	.71**	
*Mature*	.16**	.27**	−.20**	.46**	0.21
**Female Faces**					
	***Criminal***	***Threatening***	***Trustworthy***	***Dominant***	***Angry***
*Threatening*	.59**				
*Trustworthy*	−.73**	−.57**			
*Dominant*	.32**	.34**	−.12**		
*Angry*	.49**	.54**	−.80**	−.01**	
*Mature*	.14**	.30**	.03**	.56**	.03

* *p*<.05, one-tailed;

** *p*<.001, one-tailed.

## Methods

### Ethics Statement

The research protocol was reviewed and approved by the University of Leicester School of Psychology Research Ethics Committee. Written informed consent was obtained from participants prior to their participation.

### Participants

512 participants (67% Caucasian; *M* age = 30.93, *SD* = 11.40; *n* = 333 female) from the University of Leicester volunteered. 256 participants rated the mugshots and 256 rated the controlled faces.

### Materials

The naturalistic photographs (*N* = 80, 40 female) were randomly selected from an offender database housed on the Oklahoma Department of Corrections website (http://www.doc.state.ok.us/). The database allows the user to specify search criteria. The majority of participants were expected to be college age (18–25 years old) and Caucasian; therefore, race/ethnicity (Caucasian) and age (18–25 years) were entered as search criteria to control for possible effects of own-race bias and own-age bias on attention [17]. The mugshots were converted from color to black and white, and cropped such that only the person’s face was displayed. As a result, clothing and background cues that were naturally varying across the photographs were eliminated as possible sources of information that could influence person perception processes.

**Figure 2:**
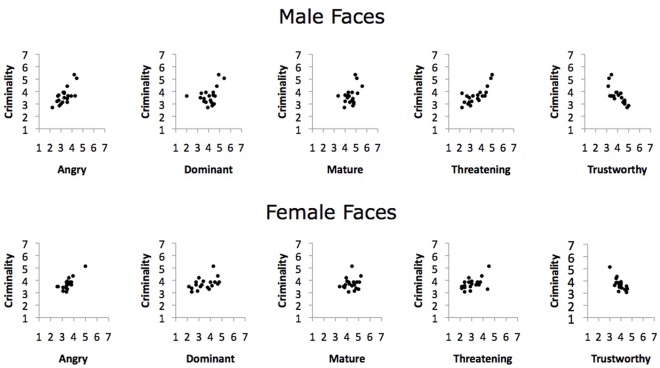
Scatterplots illustrating the bivariate relationship between criminality with the other attributes measured for the controlled photos by face gender.

**Table 3:** Pearson’s Bivariate Correlation Coefficients for Faces in Neutral Pose, Controlled Photographs.

Male Faces					
	*Criminal*	*Threatening*	*Trustworthy*	*Dominant*	*Angry*
*Threatening*	.77**				
*Trustworthy*	−.76**	−.69**			
*Dominant*	.42*	.62*	−.18		
*Angry*	.70**	.61**	−.71**	.46*	
*Mature*	.42*	.32	−.10	.35	.22
**Female Faces**					
	***Criminal***	***Threatening***	***Trustworthy***	***Dominant***	***Angry***
*Threatening*	.52*				
*Trustworthy*	−.74**	−.46*			
*Dominant*	.47*	.52*	−.24		
*Angry*	.78**	.68**	−.52*	.54**	
*Mature*	.14	.55*	−.02	.51*	.49*

* *p*<.05, one-tailed;

** *p*<.001, one-tailed.

The controlled faces were obtained from the Radboud Faces Database, which contains high quality validated face stimuli [18]. The adult actors (N = 39, 19 female) were trained to display emotions using the Facial Action Coding System. Clothing, lighting conditions, focal distance, and image background were held constant across photographs. Actors wore black t-shirts, had their hair pulled back from their faces, and wore no jewellery or glasses. Three color photographs of each actor in frontal view with the eyes directed straight ahead were obtained; each photograph depicted the actor with a different emotion display, including anger, happiness, and neutral expressions. The photos were not converted to black and white because they were originally validated in color [18].

### Measures

Each participant rated faces from either the naturalistic or the controlled emotionally neutral databases on a single attribute; participants were not told from where the photographs had been sampled. As a result, participants rating mugshots did not know they were rating mugshots or individuals who had been accused of committing a crime. Additionally, participants rated either male or female faces to avoid the possible influence of gender specific carry-over effects in making the ratings. To illustrate, female faces on average should appear arguably less dominant compared to male faces. Consequently, a female face may be given a lower dominance rating if she is rated following a male rather than a female face.

The faces were presented one at a time on a computer screen, and ratings were made with a 7-point Likert-type scale, with higher scores reflecting more of the attribute. The attributes measured for the naturalistic and emotionally neutral controlled faces included criminal appearance, trustworthiness, dominance, threatening, maturity (i.e., how baby-faced versus adult-like the person appeared), and anger. Responses were entered by the participant via a keyboard; there was no response deadline. Every attribute was rated by at least 16 participants.

**Figure 3:**
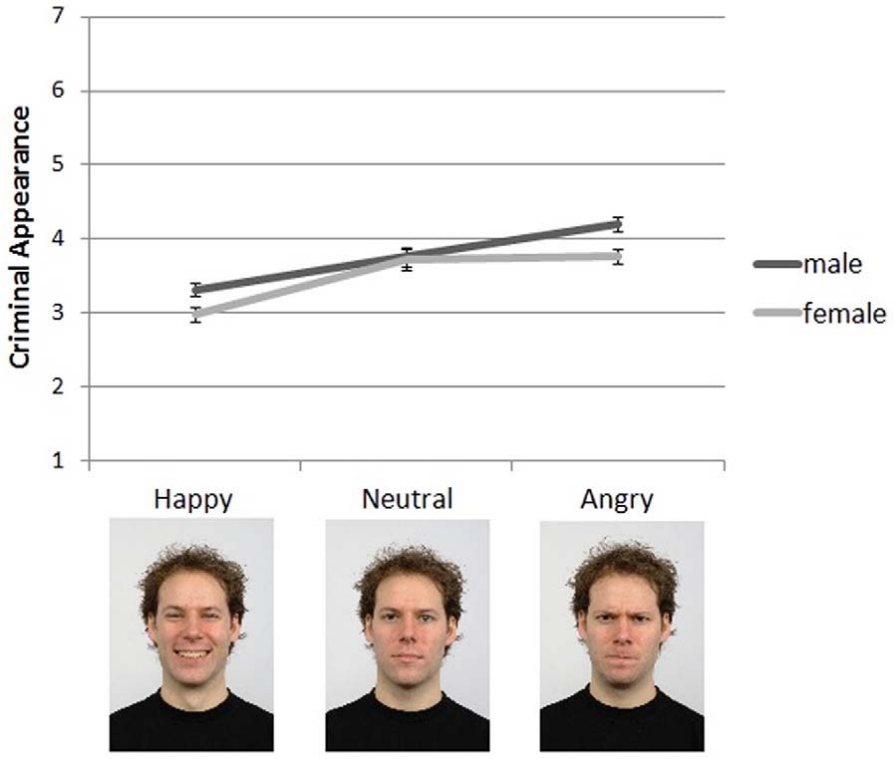
Mean (±1 *SE*) criminal appearance ratings of the controlled faces by emotional expression condition and actor gender. Examples of the male face stimuli are presented along the x-axis for each emotion condition. Female stimuli not shown; visit http://www.socsci.ru.nl:8180/RaFD2/RaFD?p=main for further information about the face stimuli.

## Results

Attribute ratings were not found to vary depending on the gender, ethnicity or age of the participant, and were reliable, as Cronbach’s alpha ranged from.88 to.98 (see Table 1). Ratings for each attribute were averaged across participants for each face. Consequently, each face had a mean value for each of the attributes measured. Mean ratings across the faces were normally distributed for each attribute. The faces served as the unit of analysis in all statistical computations. Inferential statistical results for the naturalistic photographs and the controlled photographs will be presented separately. Alpha was set to.05 in all analyses.

### Naturalistic Photographs

The first set of analyses ascertained whether the 2D model accounted for perceptions of criminality for the persons portrayed in the mugshots. Correlation matrices for the attribute ratings for males (top panel) and females (bottom panel) are given in Table 2 and scatterplots illustrating the relationship between criminality and the other attributes are given in Figure 1. As shown, trustworthiness and criminality were negatively correlated for male and female faces (*r* = –.89 and *r* = −.73, respectively, *p*’s <.001). Additionally, dominance and criminality were positively associated for males and females (*r* = .73 and *r* = .32, respectively, *p*’s <.05). As found in previous research, trustworthiness was negatively associated with ratings of anger (*r* = −.88 males and *r* = −.80 females, *p*’s <.001), and faces rated high in dominance were also rated high in maturity (*r* = .46 males and *r* = .56 females, *p*’s <.001). Finally, threat ratings were significantly associated with perceptions of criminality (*r* = .83 males and *r* = .59 females, *p*’s <.001), trustworthiness (*r* = −.89 males and *r* = −.73 females, *p*’s <.001), and dominance (*r* = .73 males and *r* = .32 females, *p*’s <.05). The latter results suggest that criminality and threat are overlapping constructs.

Multiple linear regression analysis was performed next to assess whether both trustworthiness and dominance significantly predicted criminal appearance ratings when considered together; modelling was performed separately for male and female faces. For male faces, the overall model was significant (*F*(2, 37) = 79.67, *p*<.001) and accounted for 80% of the variability in the criminality ratings (R^2^_adj_ = .80). Trustworthiness (β = −.74, *p*<.001) and dominance (β = .31, *p*<.05) each contributed significantly to the model. For female faces, the overall model was significant *F*(2, 37) = 25.90, *p*<.001) and accounted for 56% of the variability in the criminality ratings (R^2^_adj_ = .56). As was the case for the male sample of photos, trustworthiness (β = −.70, *p*<.001) and dominance (β = .23, *p*<.05) each significantly predicted criminality ratings. These results support the hypothesis that evaluations of trustworthiness and dominance underpin judgments about whether a male or female face appears criminal, supporting the conclusion that the 2D model can parsimoniously account for whether a face will be perceived as criminal-looking.

### Controlled Photographs

The results thus far are in keeping with the hypothesis that faces that look untrustworthy and dominant are more likely to be perceived as criminal. The next set of analyses assessed whether these findings could be replicated with a set of faces in which emotional expressions, background, focal distance, and clothing were controlled.

Correlation matrices for the attribute ratings made in response to the emotionally neutral photos are given in Table 3 (males and females shown in top and bottom panels, respectively) and scatterplots illustrating the relationship between criminality and the face attributes are given in Figure 2. The results were highly similar compared to those obtained with the naturalistic photos. As shown in Table 3, criminal appearance ratings were negatively associated with trustworthiness (male *r* = −.76 and female *r* = −.74), and positively associated with dominance (male *r* = .42 and female *r* = .47). Trustworthiness was negatively associated with anger (male *r* = −.71 and female *r* = −.52), and dominance was positively associated with face maturity (male *r* = .35 and female *r* = .51). Additionally, perceptions of threat were significantly associated with perceptions of criminality (male *r* = .77 and female *r* = .52), suggesting that the two constructs overlap.

Actor gender, trustworthiness ratings and dominance ratings were regressed onto the criminality ratings to test whether perceptions of criminality are accounted for by both valence and dominance. A significant model fit was produced, *F*(3, 38) = 22.05, *p*<.001, which accounted for 62% of the variability observed in the criminality ratings (R^2^_adj_ = .62). Both trustworthiness and dominance were significant predictors in the model: Criminal appearance ratings were negatively associated with trustworthiness (β = −.70, *p*<.001) and positively associated with dominance (β = .31, *p*<.01). Gender (males were coded using 1, and females 0) did not significantly contribute to the model (β = −.08, *p* = .47). In sum, these results are consistent with the conclusion that criminal perceptions can be derived from structural properties of faces that connote trustworthiness and dominance.

The final set of analyses examined the effects of overt emotional expressions on perceptions of criminality. Faces should appear more criminal when they portray a negative emotional expression. The criminal appearance ratings were submitted to a 2 (gender) x 3 (emotional expression) mixed ANOVA, with actor gender as the between groups factor and emotional expression as the within groups factor. Descriptive statistics for this analysis are presented in Figure 3. Criminal appearance ratings significantly varied depending on emotional expression, *F*(2, 74) = 44.95, *p*<.01, η_p_^2^ = .55. In keeping with prediction, happy expressions were associated with lower criminality ratings compared to neutral (*t*(38) = 6.25, *p*<.01) and angry expressions (*t*(38) = 11.14, *p*<.01). Angry faces were also rated as more criminal in appearance compared to neutral faces (*t*(38) = 2.26, *p*<.05). Additionally, a significant main effect for gender was found, *F*(1, 37) = 5.02, *p*<.05, η_p_^2^ = .12. Male actors were given higher criminal appearance ratings on average compared to female actors (*M* = 3.76 versus *M* = 3.49, respectively). Emotional expression and gender did not interact significantly. Taken together, these results are consistent with the hypothesis that perceptions of criminality are influenced by emotional expression, with faces that have angry expressions being perceived as more criminal-looking.

## Discussion

Applied social cognition research has found that people readily agree regarding whether a given face appears criminal [3], [7], and have well defined stereotypes concerning what criminals look like [1], [2], [3], [4]–[5]. The present study drew from the 2D face evaluation model [16] to make predictions about the features of faces that underlie criminal appearance. The 2D face evaluation model proposes that two dimensions–trustworthiness and dominance, can capture the spontaneous social judgments that we make about others based on their facial appearance. Previous research has indicated that faces are viewed as more threatening if they are perceived as untrustworthy or dominant, suggesting that snap judgments made from facial appearance have evolutionary adaptive value and enable us to determine whether to approach or avoid someone [16]. The present study extended this logic, predicting that criminal-looking faces would be rated as more threatening, less trustworthy and more dominant compared to other faces.

Results indicated that the 2D face evaluation model accounts for criminal appearance ratings in police mugshots and controlled photographs, as faces rated high in criminal appearance were rated as less trustworthy and more dominant. Additionally, emotionally neutral faces in the controlled sample of photos were deemed as less trustworthy if they were perceived as angry, and more dominant if they were morphologically more mature-faced. Additionally, systematically manipulating the actor’s emotional expression was found to influence criminal perceptions. Angry faces were rated as the most criminal, followed by neutral and happy faces. These findings held for both male and female faces.

Previous research has shown that compared to morphologically babyish adult faces, morphologically mature adult faces are viewed as more dominant and physically strong, and less honest, warm, and kind [11], [12], [13], [14]. What is more, faces perceived as angry are viewed as less trustworthy, and are more likely to be avoided compared to fearful faces [19]. The present study extends these past findings by demonstrating that morphological features that signal high dominance and low trustworthiness can also signal high criminality.

Previous research has found that threatening faces are perceived as less trustworthy and more dominant [16]. Criminality and threat may very well be constructs that overlap considerably. Indeed, the overarching purpose of the present study was to examine whether evaluations of criminal propensity based on facial expression could be explained by the 2D model, which heretofore has analysed threat as a general construct, under which criminality can in all likelihood be subsumed. The present study found that the shared variability (r^2^) between the criminal appearance and threat ratings was 69% for the male and 35% for the female mugshots, and 59% for the male and 27% for the female controlled photographs (see Tables 2 and 3). Whilst criminal appearance and threat are strongly related, especially for males, there seems to be scope for additional explanatory factors that account for variation in perceptions of criminal appearance. For instance, the extent to which threat and criminality overlap probably depends on the type of crime and the gender of the criminal (see [20]). Participants in the present study may have had different crimes in mind when rating the criminality of the people in the mugshots versus the controlled photographs. Criminality and threat could overlap conceivably more when people have in mind crimes against the person rather than, say, white-collar crime. The present study, like previous ones examining criminal face bias, did not provide the raters with a crime category. An interesting avenue for further exploration would be to examine whether different types of crime differentially impact perceptions of dominance and trustworthiness. For instance, trustworthiness and emotion displays might be stronger predictors of criminal appearance evaluations than facial features that connote dominance in evaluating whether a person has committed a white-collar compared to a violent crime.

This paper adds to the growing body of research finding that we make social judgments about others based on their facial appearance. On the one hand, spontaneous inferences derived from faces regarding criminal propensity may have adaptive value in that we avoid someone who is potentially threatening. On the other hand, such inferences may also influence social decision making when they should not, such as when a person is being judged in legal proceedings. The conclusion that social judgments concerning criminal propensity are tied not only to transient emotional displays, but also to morphological features of faces is sobering, as it suggests that there is little that individuals or society can do to prevent these judgments from occurring. There may be some countermeasures at hand, however. For example, warning an eyewitness, defendant, or a jury regarding the effects of facial appearance on social judgments in an effort to avoid social judgments of criminality may be one avenue worth exploring (for further discussion see [21]–[22]). Additionally, the results of this study suggest that controlling the emotional expression and matching the structural properties of lineup faces may be important in minimizing criminal face bias effects in lineups. Clearly additional research is needed to determine whether such measures can eliminate the biasing effects criminal appearance on social judgment.
